# ‘I do Things that I don’t Really Want to do …’: Understanding the Everyday Lives of Family Carers of People With Dementia

**DOI:** 10.1177/14713012251368682

**Published:** 2025-08-11

**Authors:** Jacoba Huizenga, Sascha Bolt, Jean Pierre Wilken, Nienke Bleijenberg, John Keady, Tine Van Regenmortel

**Affiliations:** 1 Research Center Social Innovation, 8119 HU University of Applied Sciences Utrecht, The Netherlands; 2 Department of Tranzo, School of Social and Behavioral Sciences, 7899 Tilburg University, The Netherlands; 3 Research Center Healthy & Sustainable Living, 8119 HU University of Applied Sciences Utrecht, The Netherlands; 4 Department Julius Center for Health Sciences and Primary Care, University Medical Center Utrecht, The Netherlands; 5 Division of Nursing, Midwifery and Social Work/Greater Manchester Mental Health NHS Foundation Trust, 5292 The University of Manchester, UK; 6 HIVA—Research Institute for Work and Society, Faculty of Social Sciences, University of Leuven, Belgium

**Keywords:** everyday life, family carers, psychosocial, dementia, neighbourhood, citizenship

## Abstract

In the Netherlands, where this study was conducted, there are around 800,000 family carers of people with dementia. Research into the needs and priorities of people with dementia and their family carers is crucial for developing tailored care and meaningful support. However, current research lacks attention to the everyday life experiences of caring for someone with dementia at home. Therefore, the research question this study aimed to address was: how do family carers of people with dementia living at home approach and experience their everyday life in a caring context? The study used a qualitative design, underpinned by a phenomenological approach. 15 family carers (10 partners and five adult children) participated in open interviews. Thematic analysis was used to document and structure the data. A member check was performed on the emergent findings through a focus group with six family carers (all care partners). This process resulted in four discrete but interlinked themes that reflected how family carers approach and experience caring at home for a person with dementia, namely: (1) Finding and keeping routines that work; (2) Focussing on small moments; (3) Rebalancing connections; and (4) Thinking ahead. These themes also emphasise the unfolding nature of everyday life that is constantly changing for family carers.

## Introduction

Given the projected trends in population ageing, the number of people with dementia is expected to increase rapidly (Nichols et al., 2019). Current estimates indicate that 46.8 million people worldwide currently have dementia, a number projected to climb to 74.7 million by 2030 and 131.5 million by 2050 (Patterson, 2018). Approximately two-thirds of individuals with dementia reside in the community, where they are mostly supported by family carers. In the Netherlands, there are 300,000 people with dementia and around 800,000 family carers, 70% of whom are women (Francke et al., 2024). Caring for a person with dementia can have profound and far-reaching consequences for these family carers (Reckrey et al., 2021).

Dementia care is often criticised for not aligning well with what is important to people with dementia and their family carers, leading to suboptimal care and support (McDermott et al., 2018; Morrisby et al., 2019; Reilly et al., 2020). Many studies have found that family carers of people with dementia face a range of issues, including the psychological and emotional challenges of the caregiving experience (Lindeza et al., 2024; Macdonald et al., 2019), receiving inadequate support services (Bannon et al., 2022; Mercogliano et al., 2024), and having to develop a range of coping strategies to successfully navigate everyday life at home (Balasubramanian et al., 2024; Gilhooly et al., 2016; Li et al., 2021; Zhang et al., 2024). In contrast, other studies have focused on the experiences of, and changes in, spousal relationships (Egilstrod et al., 2018; Hellström & Torres, 2021; Pozzebon et al., 2016), the dyadic relationship between the family carer and the person with dementia (Hochgraeber et al., 2023), and broader family dynamics (Smith et al., 2022; Zhang et al., 2024) including the notion of couplehood (Bielsten et al., 2018; Dunn et al., 2024; Hellström et al., 2007; Hellström & Torres, 2021; Swall et al., 2020). Additionally, research into care dynamics and family network perspectives has contributed to a deeper understanding of the complex interactions and support structures within families caring for someone with dementia (Esandi et al., 2021; Nagl-Cupal et al., 2024; Neubert et al., 2022 Nagl-Cupal et al., 2024; Neubert et al., 2022).

In contrast to this focus on psychosocial research, the concept of ‘everyday life’, as conceptualised in the sociological literature, refers to routine and familiar activities that shape an individual’s understanding of themselves, others, and society (Scott, 2009; Sztompka, 2008). The sociology of everyday life places emphasis on the mundane, ordinary, and often overlooked aspects of how people navigate and make sense of their lives (Scott, 2020). With this context in mind, sociological research has both developed and highlighted critical social perspectives on living with dementia (Bartlett & Lid, 2025; Collins & Fletcher, 2024), including an emphasis on human rights (Cahill, 2020) and the citizenship approach (Bartlett & O’Connor, 2007). These approaches call for an agenda framed around everyday life as a way of extending understanding and framing the lived experience of dementia (see also: Huizenga et al., 2022, 2023).

The sociology of everyday life therefore seeks to understand how people with dementia and their family carers engage with the social world and navigate their day-to-day lives, highlighting the social, relational, and contextual nature of living with dementia (Kalekin-Fishman, 2013; Nedlund et al., 2017). Yet, to date, this perspective has received relatively little attention in the literature. To address this deficit, the current study builds on two previous studies that focused on the everyday life from the perspective of people with dementia (Huizenga et al., 2022, 2023). This paper focuses on family carers, using the same lens of everyday life. The research question that this study aims to explore is: how do family carers of people with dementia living at home approach and experience their everyday life in a caring context?

## Methods

### Design

This study used a qualitative design to gain an in-depth understanding of family carer’s perspectives on their everyday life with a person with dementia, underpinned by a phenomenological approach (Schutz, 1973).

### Participants and Recruitment

The sample was recruited in the Netherlands, with most participants coming from the central provinces. Open, individual interviews were held in the participants’ homes or at another location of the family carers’ choice. Participants were purposively selected family carers based on having a caring relationship with a person with a diagnosis of Mild Cognitive Impairment (MCI) or dementia, either self-reported or by a professional assessment. The research team aimed to include a diverse sample in terms of the nature of the relationship to the person with dementia (partner or adult child), gender, educational background, and country of birth. The study specifically focused on family carers of people with dementia living at home. Some participants had a relative who was in the process of moving to a care home at the time of the interview whilst others were invited to join this study by the first author (JH) because of their previous exposure to another study. The remaining sample were recruited by contacting local healthcare organizations and professionals to invite potential participants to consider joining the study by means of an invitation letter.

Throughout the text, family carers are referred to as ‘participants’. Explicit reference is made to the nature of their relationship to the person with dementia (partner or son/daughter) when appropriate. The term ‘partner’ may refer to a marital partner or spouse, or to a significant other/life companion.

### Data Collection

Interviews were conducted by the first author (JH) between May 2022 and June 2024. Participants received information about the study’s aims before giving consent and they could withdraw at any time. In five cases, the first author (JH) started by interviewing the person with dementia. These interviews were used in a separate study. During a second appointment, the focus shifted to the family carer. In two of these cases, a third appointment was required to complete the interview. The first author (JH) was therefore flexible in adapting to what worked best in each situation. The interviews lasted between 60 and 90 minutes.

The interviews always began with the question, “How do you see the everyday life with your partner/parent with dementia?” This question served as a starting point for participants to naturally share their experiences. Follow-up questions were inspired by the everyday life domains that came to the fore in the extensive work on everyday life (Huizenga et al., 2022, 2023), such as activities, relationships and the environment. Participants were also encouraged to share additional topics that were meaningful to them. The first author (JH) conducted all interviews, while the second author (SB) joined in reflecting on transcripts during data collection for deepening understanding. Audio recordings of interviews were transcribed verbatim, and all names used in this article are pseudonyms in line with the study protocol.

### Data Analysis

A thematic analysis was conducted to examine the data (Braun & Clarke, 2022), using the everyday life themes identified by Huizenga et al. (2022; 2023) as sensitising concepts to guide the initial coding of the transcripts. A coding tree was developed through an iterative process of moving back and forth between the transcripts and codes. During this reflexive phase, the first author (JH) and second author (SB) independently coded eight interviews and the first author coded all interviews to determine if codes adequately covered all the data. Gradually, the codes were modified through reflection and discussion between the authors. This inductive approach also resulted in the development of different themes that more accurately depicted the way the participants, i.e. family carers, approach and experience everyday life. The final coding tree can be found in the Supplemental Material. The software program Atlas.ti was used to support data management of data and codes. During the analysis the first author (JH) kept a reflexive journal noting down assumptions, thoughts, and emerging insights. The reflexive journal was also used as supportive data in reporting the findings from the study. Emergent findings and themes were regularly discussed with all authors for consensus and triangulation (Carter et al., 2014).

To ensure the authenticity and credibility of the emergent themes for participants, the findings were cross-checked through a focus group interview during the data analysis process. A professional from a meeting centre invited family carers, all of them were partners aged between 69 and 84 years, caring for persons with different forms of dementia (Alzheimer’s disease, vascular dementia, and frontotemporal dementia) with caring experiences ranging from 1 to 14 years. The emergent findings were presented to the group, and they were invited to provide input based on their perspective on everyday life. The duration of the focus group meeting was 69 minutes. The meeting was recorded and transcribed. Initially, the transcript was coded in the same manner as the interviews. During the finalisation of the themes, this transcript was used to identify relevant insights from the focus group to enhance the descriptions of the themes.

### Ethical Considerations

The study followed The Dementia Enquirers Gold Standards for Ethical Research (Innovations in Dementia, 2023). All participants gave informed consent for participation and use of data for this study. The study protocol was approved by the Research Ethics Committee of HU University of Applied Sciences (reference number 2022-5).

### Research Team and Reflexivity

The first author (JH) is a PhD candidate. She is a psychologist with clinical experience and has experience as lecturer in Social Work. She also brings personal experience as family carer, as her father had dementia, and her mother was diagnosed during the research. The rest of the authors have interdisciplinary academic backgrounds in social work, social sciences and (mental health or community care) nursing.

## Findings

As shown in Table 1, 15 participants took part in the study. They were 10 partners and five adult children, nine women and six men, with diverse education backgrounds. One participant had a migration background. The type of dementia was mostly Alzheimer’s disease, although some did not explicitly disclose the type but mentioned ‘dementia’. There was a variety in the time since (official or self-reported) diagnosis.

**Table 1.** Participant Characteristics

Characteristics	*n (N = 15)*
Gender	
Female	9
Male	6
Relationship with the person with dementia	
Partner	10
Child	5
Living situation of the person of dementia	
Alone	2
With partner	13
Diagnosis	
MCI	2
Alzheimer’s disease	9
Parkinson’s Dementia	1
Not specified	3
Country of origin	
The Netherlands	14
Country in Eastern Europe	1

The analytical process resulted in the formulation of four discrete but interlinked themes: (1) Finding and keeping routines that work; (2) Focusing on small moments; (3) Rebalancing connections; and (4) Thinking ahead. Each theme will now be described in turn; the theme numbering is not intended to be hierarchical.

### Theme 1: Finding and Keeping Routines that Work

Participants experienced dementia as a disruption of the mundane routines of everyday life. They shared that upholding routines became increasingly challenging for the person with dementia because of forgetfulness, decreased understanding of how to perform the routines and lacking overview of tasks, as Rob explained:

“She [partner] is busy with three things at once and then she doesn't remember what she was supposed to do. I have to repeat myself, telling her to do that and nothing else. Then she walks away and is just busy with two other things.”

To deal with these everyday disruptions, participants instinctively looked for ways to keep the routines going. For instance, they used concrete and short directives, and tried to reduce of their expectations. The disruption of routines was deeply affecting as the ‘normal’ no longer appeared ‘normal’ and was in a constant state of flux. Participants also tried to maintain order in the day, such as through the use of schedules, to ensure consistency in activities. Practical ‘tools’ like agendas and smartphones, were utilised to support these activities. As an illustration, Mies (partner) shared about a special clock positioned in the living room and into which their son programmed the day’s activities, accompanied by personal messages for his parents to follow.

Another notable disruption of the routines described by participants concerned sleep-related issues. Some persons with dementia got out of bed several times during the night to use the toilet and subsequently became disoriented. Other issues mentioned included being woken up by lights being turned on in the middle of the night, needing assistance with physical problems, or experiencing a disturbed day–night rhythm as Rob (partner) explained:

“It’s also been half past five, and then she really pushes through like a train. “I have to go to [name daycare].” And if I go against it: “I have to go to [name daycare]!” Please, stop it. She puts on her bathrobe and starts cooking porridge. I’m losing my mind.”

Whilst many household chores were gradually taken over by participants, some recognised the importance of the person with dementia continuing to contribute to everyday life. This often meant slower progress in dealing with the everyday tasks and in risking unexpected situations arising, such as having dirty dishes placed in the cupboard instead of first being cleaned and dried. Such mundane events frequently caused frustration, especially when participants had to solve them on their own. Participants also continued to seek alternative ways of ‘getting things done’ by automating tasks, such as using a robot lawnmower. At the same time, participants felt that they must always be ‘on guard’ to prevent things from going wrong, such as with cooking. Consequently, for participants, everyday life revolved more and more around the routines of the home, a situation that also existed for arranged support including from their own adult child(ren) where this was present. In contrast, when adult children did not see their parent(s) every day but wanted to remain connected, they often remained in contact with neighbours as a proxy for meaningful everyday involvement.

Participants believed that keeping the person with dementia active during the day was essential. They described this as a need for stimulation and to keep the person with dementia’s brain working for as long as possible. However, motivating the person with dementia to uphold these daily routines could be challenging, as Lisa (daughter) explained:

“If you suggest: ‘Oh, you always enjoyed doing that....’. ‘You don’t know what it’s like to be 80 and have pain in your feet, then you wouldn’t go for a walk either.’ Then I think: it’s actually good to go for a walk even if your feet hurt.”

To create a structure to the day, most participants in the sample looked for new activities for the person with dementia, such as attendance at daycare. Some participants also felt encouraged and supported in finding suitable daycare by family and professionals. Most participants in the sample had experience with organised day activities for the person with dementia, and it was mostly reported as being enjoyable but challenging if the relative did not want to go. The opportunity for participants to find time and personal space within the everyday demands of caring was important, as this explanation by Mies suggests:

“That day [daycare attendance by the person with dementia] is for me. You just need it because constantly... I keep an eye on you. And now I have to let you go for a day. And that is probably good for both sides.”

Whilst organised day activities or respite care allowed participants to have more time for themselves, this was not always sufficient. For example, Loes expressed her discontent because she wanted to go to a club in the evening, but her husband could not attend the day activity centre at that time as it was closed, thus preventing her from going to her club.

In a similar vein, losing the ability to drive for the person with dementia often represented a prominent disruption in everyday routines, especially when the family carer had to take over the driving responsibilities. Whilst some people with dementia in the sample used a bicycle or public transport instead, everyday life for participants was complicated by poor connections in public transport and/or unreliable timetables. Moreover, obtaining compensation for public transport could also be problematic:

“I pay 18 euros for a card, and he goes to the care farm twice. The case manager was here and then I said: ‘Maybe I can get a reimbursement?’ Well, that is not happening at all. Is there no other fund where you can go? I don’t know where to go. That makes me so tired.” (Ria, partner)

Finding and keeping routines that worked for participants to support the person with dementia required constant adaptation and attention. Assistance from other family members, daycare, or respite care can support these everyday routines and allow participants to reclaim part of their lives outside of caring responsibilities.

### Theme 2. Focusing on Small Moments

In seeing life as a series of small moments, participants emphasised the importance of leisure activities or volunteering for a sense of joy and purpose for the person with dementia. In everyday life they tried to maintain long-standing activities or find new ones. Some participants arranged adjustments, such as singing in a choir, without participating in concerts themselves. Participants also valued joint activities, like going on holiday together and doing day trips:

“Then we cycle together. We really enjoy cycling. Then you go for a drink, we take a cooler with us. You can go anywhere here, it’s so beautiful.” (Inge, partner)

Participants valued the presence of friends of the person with dementia, and some old friends were still in contact. They found peers with dementia helpful for their relative and for themselves.

“He really hopes to have found a buddy. He also loves cycling. She has also just stopped working. We visited there last week, and it clicked very well.” (Maaike, partner)

Participants also appreciated small acts of kindness, such as when someone else helped the person with dementia in a grocery store. This provided a feeling of being seen.

Another way of focusing on the everyday small moments was by making the house cosy and creating a pleasant garden or balcony space, for example with flowers or a bird box. This created a sense of home. As the data revealed, meaningful objects in the house were a way of remembering and reliving good memories with participants referring to photographs of significant others or past events in their lives. Reflecting on past accomplishments in education and work and emphasising their roles as parents or grandparents also evoked proud feelings and small moments of connection. Others enjoyed reminiscing over collected stones, art, or tiles with sayings, with some objects made by (grand)children, or by themselves, as Ria (partner) shared:

“I embroidered this [pointing to a tile]: what you do with love always goes well.”

For a few participants, focusing on small moments in everyday life was more challenging as they mentioned how everyday existence had been ‘tough’ for them, especially for those caring for people with more advanced dementia. Despite these challenges, everyday small moments were experienced by participants, such as a visit of a grandchild, having a meal together, holding hands, and walking the dog in the park.

### Theme 3. Rebalancing Connections

Most participants living together with a partner expressed a strong desire to stay connected with the person with dementia, driven by deep affection and loyalty. They wanted to live in the house together for as long as possible:

“We have endured 50 years together. We should be able to manage these last few years as well.” (Dirk, partner)

Some partners struggled with the feeling that they had ‘lost’ their soul mate, become a carer, and had to take on more and more responsibilities in their everyday life. That said, Maaike (partner) mentioned she was not happy with the Dutch term ‘mantelzorger’ (translated literally as ‘cloak carer’) as it emphasised her role as caregiver whilst she also identified herself as partner.

Participants noticed that the person with dementia became less able to attune to others. In everyday life, participants, living together as partners, experienced communication between them and their partner with dementia in a constant state of change, such as when the person with dementia insisted that they were ‘right’ or had unforeseen outbursts of anger. In many ways, their previous ways of interactingno longer worked, challenging them to act cautiously in their everyday communication, as Loes (partner) explained:

“It’s always a matter of weighing things up, like choose your battle. What do you let go and what is important, and do you stand your ground?”

Discussing everyday communication issues with the person with dementia was experienced as difficult, as this was often perceived as ‘offensive criticism’ by the person with dementia. Some partners mentioned occasions of verbal and/or physical aggression, as Inge (partner) shared:

“At a certain point, I can’t take it anymore, then I get angry, and we argue. A lot of arguments, really. I can’t handle it anymore. Sometimes I don’t say anything to him for almost an entire day. Then I think, you deal with it yourself.”

Another challenge was found in dealing with the surfacing of past experiences of the person with dementia, as Rick (partner) explained:

“She lost her mother at the age of eleven. She didn't have a normal childhood. She thinks: I always took care of my father and mother in the past. You hear that five or six times a day: “What did I do to deserve this? I have always taken such good care of everyone.”

Dementia influences relationships in the family as it requires an act of caring for one another. Some of the participants shared that this interaction drew them together as a family, and they managed to emotionally support each other, as Hanneke (partner) shared:

“That I don’t have to do it all alone. We are a very close family.”

Others missed the support or felt guilty about putting too much pressure on their adult children. Adult children themselves could experience their relationship with siblings as collaborative, whilst others missed their siblings’ support and felt the need to push for more equal responsibilities.

The person with dementia may express needs that the adult child cannot or does not want to fulfill, especially as caregiving becomes more demanding. This dilemma was highlighted by Lisa (daughter):

“I feel like I have to do it. I’m not really a caring type. I do things that I don’t really want to do. Actually, she needs more and maybe also from me. Then I notice that I can’t handle it myself. It’s not just the lack of time but also the emotional strain.”

Family relationships could also be strained due to physical distance or earlier conflicts that are lived out in the everyday. The experience of living with dementia puts pressure on the existing family dynamics, especially in case of painful experiences in the past. Such shifting dynamics can trigger feelings of trauma and resentment:

“She had that in the past as well. She has lived through tough times in her childhood. And how it was for us, she didn’t notice. She became a widow quite young. That we lost our father, she had it difficult with. It's the same now. She suffers.” (Lisa, daughter)

Participants also mentioned the importance of friends and neighbours. Some partners noticed that friends had become more reluctant to connect with them as a couple since one of the partners had dementia and was less able to communicate. As a result, friends visited them less frequently. Others missed a sense of connection in the neighborhood and felt isolated. Living in a care apartment centre with more people of their own age was mentioned as being helpful, as the building facilitated an opportunity to meet others.

Constantly adapting to the changes brought by dementia meant rebalancing the existing and evolving dynamics in the relationship between the family carer and the person with dementia, as well as within the wider social network. These dynamics are at play in everyday interactions.

### Theme 4. Thinking ahead

Participants viewed the dementia diagnosis as predictive of a certain future. They expected that the person with dementia would ‘deteriorate’, quickly or slowly, resulting in an inability to stay at home and ultimately leading to death. Most participants worried about the progression of the disease and its impact on them, as Rachel (partner) explained:

“The greatest thing I worry about, we often talk about, what would happen if he got really ill or I can’t look after him still. Nowadays I am thinking about what I will do when he gets to a point his behaviour is, let’s say so bad that I can’t do anything. I can’t deal with him? Then what do I do?”

Participants arranged daycare or respite care for the person with dementia to be able to continue caring in the future (as also discussed in Theme 1). They also anticipated a future move to a home with more care, either for the person with dementia or for themselves as a couple. Participants felt the need to involve the person with dementia in this process, for example by visiting care homes together. The thought of being separated as partners in case of a future move to a care home triggered sadness for some, but a relief for others. At the same time, partners felt sorry for the person with dementia, who may feel sad at the thought of separation. Knowing that a move is inevitable, some participants started preparing for the future in the here and now by tidying up their homes or by envisioning their new ‘home’. Interestingly, meaningful objects could also serve as a way of thinking ahead and planning a new home, as Rick (partner) explained:

“If there is space, someone will come to check the house situation about four weeks in advance. Then you will get an indication of what items you can take from home that are important to her. So she could bring her own chair, a television cabinet.”

In obtaining more intensive forms of care, participants found the bureaucracy of the care system daunting and difficult to assimilate into their everyday life. They struggled to understand forms and letters and found that seeking assistance required significant time and effort. This challenge intensified when participants felt that caregiving became too burdensome, and the person with dementia could no longer live at home. The waiting and uncertainty created tension and worry about the future. Participants often felt unheard in their need for help, leading to a strong sense of powerlessness as Ria (partner) shared:

“The case manager cannot decide or arrange anything. Everything must go through various channels. They also say, yes, you are not urgent enough, but they only see us once every six weeks for an hour. That is very different from being in it and dealing with it all day long.”

A few participants mentioned the current emphasis in Dutch society on living at home longer due to a lack of care staff. As a result, they felt distrust towards the government, as Gert (son) explained:

“Nice that the government lets everyone live at home. But, people who live at home with someone like that, you also help them go to ruin. So, it only costs more money in the long run.”

Living longer at home can create crisis situations. During the interviews, two participants were in the process of a forced move to a care home for the person with dementia. The regulations surrounding this were considered stressful and impacting on the efficacy of everyday decision-making and routines. Indeed, participants found it difficult that the person with dementia could not comprehend the situation and that a lawyer was needed to make the final decision.

Discussing the future was not always straightforward, and talking about the end of life was particularly challenging. Some participants shared that they took the person with dementia’s end of life ‘as it comes’, whilst others shared that the person with dementia explicitly signed for ‘do not resuscitate’ or euthanasia should the need arise. For a few participants, there was also a profound sense of hopelessness about the future, and they expressed a wish for an earlier end of life for the person with dementia, like Rick (partner) shared:

“We had a conversation with the case manager about the nursing home, with the daughters present. The first reaction was: “Then I’d rather die, then I’ll just take a few pills.” We discussed it with the children, who said: “Let's just give her a pill, so that she, so to speak, is no longer here tomorrow. Then we will have peace.”

In thinking ahead, a future sense of uncertainty, and the need to be in control, is experienced as a permeating reality of everyday life. Participants valued support from other family members and professionals who genuinely understood the situation and who acted proactively.

## Discussion

This study identified four main themes in the everyday life of family carers of people with dementia: (1) Finding and keeping routines that work; (2) Focusing on small moments; (3) Rebalancing connections; and (4) Thinking ahead. These themes are discrete yet interlinked, reflecting the dynamic processes at play. Everyday life for family carers unfolds moment by moment, involving constant negotiation and adaptation to creating routines that work and make the unfamiliar, familiar.

This study, like previous research on everyday life of people living with dementia (Huizenga et al., 2022, 2023), shows that using the lens of everyday life opens a wider canvas for understanding the experience of living with dementia (Nedlund et al., 2017). It also highlights family carers’ caregiving actions in daily life, e.g. how they strive to maintain routines for the person with dementia and for themselves. Specifically, this study strengthens previous findings that indicate that focusing on the mundane is essential in the life of persons living with dementia or other disabilities and their families (Giebel & Sutcliffe, 2018; Gjermestad et al., 2017; Milligan, 2019; Ziegert, 2011). The notion of the ‘everyday’ highlights the importance of the ordinary in human existence (Pink, 2012) and family carers in this study gave insight into their everyday routines and challenges, and how they made sense of their own lived experience. Such information may not always surface when using a healthcare lens, even though care and support provided to people with dementia and their families ideally aligns with their everyday lives.

Disruptions to the mundane due to the presence of dementia in everyday life challenges the ability of family carers to participate in seemingly ordinary everyday routines and activities, both day and night. Family carers in this study felt that they were continuously adapting to the situation and to create a safe environment for care and everyday life to continue. This requires creativity, as well as finding resources within their social and connected neighbourhood network (Keady, 2024). As Neal and Murji (2015) explain, everyday life is situated in social relations and practices, where family carers act within dynamic relationship networks. The lens of everyday life attempts to capture the mundane and the routines in social relations, and by doing so, it illuminates how these social practices are dynamic, challenging, ambivalent, and always changing. Moreover, these social practices, situated within a neighbourhood and society, may also be understood through a citizenship lens. The perspective of citizenship emphasises daily social practices as a means by which individuals connect with their fellow citizens and engage with the wider community and society (Bartlett & O’Connor, 2007; O’Connor et al., 2022). Maintaining citizenship also involves macro structures, as seen in this study, that shape the family care experience (Birt et al., 2017; Collins & Fletcher, 2024). This means that supporting everyday life for family carers is not only the task of individual support workers but also a collective responsibility of communities.

This study shows that the appreciation of ‘small moments’ can make everyday life worth living. Similarly, previous studies found that caring experiences may involve feelings of gratitude and meaning, as well as strengthening relationships (Lindeza et al., 2024; McGee et al., 2024a, 2024b). This nuanced perspective differs from viewing dementia solely as a narrative of loss, which predominates in Dutch and Western media beard (Beard, 2017; Osch, 2024; Putland & Brookes, 2024a, 2024b). As is seen in this study, family carers do focus on small moments and making everyday decisions to maximise opportunities to continue to live at home. Living in the ‘here and now’ with everyday disruptions and valuing good moments is partly reflected in the dual process model of grief (Stroebe & Schut, 1999), previously mentioned in studies on family carers of people with dementia (Colquhoun et al., 2019; Merrick et al., 2016; Robinson et al., 2005). The dual process model describes a loss orientation, looking back at what is lost, and a restoration orientation, looking towards the future. This study adds that these experiences are not merely about looking back and forward but about actively engaging with all dimensions of time – past, present and future (as described by Eriksen et al., 2021), and especially the importance and value of being ‘in the moment’ (Keady et al., 2022).

When comparing the everyday experiences of family carers to those of people with dementia, carers are more prone to be thinking ahead as a part of their everyday life. These different ways of looking to the future is in line with previous research (Bolt et al., 2021). Family carers adapt contiuously to the different phases of dementia, each presenting new challenges (Clemmensen et al., 2019; Kokorelias et al., 2022). They navigate these challenges to maintain the best possible home environment, striving for this goal together with the person with dementia (Köhler et al., 2021). This study highlights the difficulty of anticipating future separation, whether by death or moving to a nursing home. Family carers are alert to their boundaries, considering when their limit of care will be reached and desiring to make informed decisions. Thinking ahead and anticipating are crucial parts of everyday life for carers of people with dementia. This is not only practical or care planning, often referred to in the literature as advanced care planning (Rietjens et al., 2017), but focuses fundamentally on what matters now and in the future (Rickstal et al., 2022). This resonates with the concept of ‘anticipatory care’ of Bowers (1987) who noted that the emotional investment in care is the most stressful aspect for family carers of people with dementia, even more so than the physical tasks involved.

Care, from a sociological perspective, is something we ‘do’ in everyday life (Ursin, 2020), i.e. we engage in social interactions and relationships with others. This study reveals the complex dynamics of rebalancing the giving and receiving in relationships (Meiden et al., 2020) when dementia enters family life. Understanding the existing relational dynamics, especially when there are intergenerational wounds, is crucial to comprehend how dementia interacts with these dynamics (Esandi et al., 2018). As Ceci et al. (2020) noted, there is much more to consider than just dementia itself.

### Limitations

A limitation of the study is that the sample included only one non-Western family carer, which may not fully represent the experiences of family carers with a migration background. As the participant in this study expressed, migration influenced the social network due to geographic dislocation and the experience of culturally insensitive care workers, which is also described in a systematic review on experiences of migrant informal caregivers (Stenberg & Hjelm, 2023). Furthermore, there is evidence that non-Western family carers have different experiences because of different values (Antoniades et al., 2025; Ar & Karanci, 2019; Duangjina et al., 2025; Nurunnaher et al., 2023). Further exploration of the way people with different cultural backgrounds approach and experience everyday life would be valuable. Another limitation of this study is that the experiences of partners and adult children were not differentiated. However, the four themes apply to both groups in this study. Future research may focus on how family carers with adverse childhood experiences approach everyday life in caring for the person with dementia. Additionally, the study did not include family carers other than partners or children. There is some research on the experiences of extended family carers (Roberto & Savla, 2022) and carers who are not (close) family, such as friends, neighbours, and siblings (Lowers et al., 2024). Changes in family structures, such as decreasing family size, divorce, and families living further apart geographically, are impacting the availability of immediate family. Notably, one in three people with dementia now lives alone (Lowers et al., 2024). Further research could explore how non-kin caregivers approach everyday life in caring for someone with dementia.

### Implications

This study highlights the everyday experiences of being a family carer of someone with dementia and how they navigate this. Incorporating the concept of everyday citizenship into care and support for families living with dementia could be valuable, facilitating support to maintain established and meaningful everyday routines like going to a grocery store (Collins & Fletcher, 2024). Embedding the everyday within the scope of professionals in healthcare and social work may enhance their capacity to foster citizenship, even if the support seems ‘ordinary’ to some. While practical support is crucial, addressing everyday life issues, including emotional and relational challenges, is equally important. Furthermore, working from an everyday life perspective on a societal level in developing dementia inclusive of neighbourhood living and practice, is valuable.

Relationships and family dynamics deserve attention in care and support. Further research is needed to explore how family carers collaborate as a network and interact with professionals (Wittenberg et al., 2024). This will provide a more comprehensive understanding of the support systems required for family carers of people with dementia. Furthermore, for future research, it could be valuable to combine and compare the perspectives on everyday life of people with dementia (Huizenga et al., 2023) and the perspectives of family carers. Specifically, future research could involve mapping the everyday life of people with dementia and their households over time to see if the identified themes continue to have meaning and how they might start to interact with one another.

## Conclusion

Four themes are identified that characterise the way family carers of someone with dementia approach and experience everyday life: finding and keeping routines that work, focussing on small moments, rebalancing connections and thinking ahead. Moreover, the study’s findings underscore the value of applying the lens of ‘everyday life’ to understand the experiences of family carers. This conceptual framing enables a shift away from deficit-based perspectives that view dementia solely through the lens of loss and decline. Instead, it opens up possibilities for recognising the agency of family carers as they creatively adapt to the ever-evolving challenges posed by dementia. The four themes hold potential to integrate and apply person and relationship-centred support for family carers.

## Supplemental Material


Supplemental Material - ‘I do Things that I don’t Really Want to do …’: Understanding the Everyday Lives of Family Carers of People With Dementia
Supplemental Material for ‘I do Things that I don’t Really Want to do …’: Understanding the Everyday Lives of Family Carers of People With Dementia by Jacoba Huizenga, Sascha Bolt, Jean Pierre Wilken, Nienke Bleijenberg, John Keady, and Tine Van Regenmortel in Dementia

## Data Availability

The data that support the findings of this study are available on request from the corresponding author. The data are not publicly available due to privacy or ethical restrictions.*
