# Supplementary material for: ‘I do Things that I don’t Really Want to do …’: Understanding the Everyday Lives of Family Carers of People With Dementia
Source: Dementia (London). 2025 Aug 11;25(6):1210–28. doi: 10.1177/14713012251368682 (PMC13304901; doi:10.1177/14713012251368682)
Supplement: Supplemental Material - ‘I do Things that I don’t Really Want to do …’: Understanding the Everyday Lives of Family Carers of People With Dementia [file sj-pdf-1-dem-10.1177_14713012251368682.pdf]

Coding tree

1. Finding and keeping routines that work
    a. Everyday routines
    b. Household chores
    c. Day activities
    d. Transport
2. Focusing on small moments
    a. Leisure activities
    b. Moments of connections
    c. House and meaningful objects
3. Rebalancing connections
    a. Relationship with a partner
    b. Relationships within the family
    c. Relationships with friends and neighbours
4. Thinking ahead
    a. Thinking about the future
    b. Anticipating the future
    c. Support of professionals and society
    d. Crisis
    e. End of life
